# Zika virus infection in the Veterans Health Administration (VHA), 2015-2016

**DOI:** 10.1371/journal.pntd.0006416

**Published:** 2018-05-24

**Authors:** Patricia L. Schirmer, Aaron Wendelboe, Cynthia A. Lucero-Obusan, Russell A. Ryono, Mark A. Winters, Gina Oda, Mirsonia Martinez, Sonia Saavedra, Mark Holodniy

**Affiliations:** 1 Public Health Surveillance & Research, Department of Veterans Affairs, Washington, DC, United States of America; 2 University of Oklahoma Health Sciences Center, Oklahoma City, Oklahoma, United States of America; 3 Veterans Affairs Palo Alto Health Care System, Palo Alto, California, United States of America; 4 Division of Infectious Diseases and Geographic Medicine, Stanford University, Stanford, California, United States of America; 5 VA Caribbean Health Care System, San Juan, Puerto Rico; Institute of Tropical Medicine (NEKKEN), Nagasaki University, JAPAN

## Abstract

**Background:**

Zika virus (ZIKV) is an important flavivirus infection. Although ZIKV infection is rarely fatal, risk for severe disease in adults is not well described. Our objective was to describe the spectrum of illness in U.S. Veterans with ZIKV infection.

**Methodology:**

Case series study including patients with laboratory-confirmed or presumed positive ZIKV infection in all Veterans Health Administration (VHA) medical centers. Adjusted odds ratios of clinical variables associated with hospitalization and neurologic complications was performed.

**Principal findings:**

Of 1,538 patients tested between 12/2015-10/2016 and observed through 3/2017, 736 (48%) were RT-PCR or confirmed IgM positive; 655 (89%) were male, and 683 (93%) from VA Caribbean Healthcare System (VACHCS). Ninety-four (13%) were hospitalized, 91 (12%) in the VACHCS. Nineteen (3%) died after ZIKV infection. Hospitalization was associated with increased Charlson co-morbidity index (adjusted odds ratio [OR] 1.2; 95% confidence interval [CI], 1.1–1.3), underlying connective tissue disease (OR, 29.5; CI, 3.6–244.7), congestive heart failure (OR, 6; CI, 2–18.5), dementia (OR, 3.6; CI, 1.1–11.2), neurologic symptom presentation (OR, 3.9; CI, 1.7–9.2), leukocytosis (OR, 11.8; CI, 4.5–31), thrombocytopenia (OR, 7.8; CI, 3.3–18.6), acute kidney injury (OR, 28.9; CI, 5.8–145.1), or using glucocorticoids within 30 days of testing (OR, 13.3; CI 1.3–133). Patients presenting with rash were less likely to be hospitalized (OR, 0.29; CI, 0.13–0.66). Risk for neurologic complications increased with hospitalization (OR, 5.9; CI 2.9–12.2), cerebrovascular disease (OR 4.9; CI 1.7–14.4), and dementia (OR 2.8; CI 1.2–6.6).

**Conclusion:**

Older Veterans with multiple comorbidities or presenting with neurologic symptoms were at increased risk for hospitalization and neurological complications after ZIKV infection.

## Author summary

Zika virus (ZIKV) infection has become an important flavivirus infection that affected over a half of a million people in the Western Hemisphere by the end of 2016. Here we show risk factors for hospitalizations and neurologic complications in a US Veteran population. Over 700 Veterans with confirmed or presumed positive ZIKV were included. Our study showed that older Veterans with multiple comorbidities and those presenting with neurologic symptoms were more likely to be hospitalized, while if a patient presented with a rash they were less likely to be hospitalized. Neurologic complications were more likely in those hospitalized or those with a prior history of a cerebrovascular disease or dementia. Better understanding of those patients most at risk for severe disease can help providers when evaluating and treating patients with ZIKV infection.

## Introduction

Zika virus (ZIKV) is a flavivirus transmitted primarily by *Aedes* species mosquitoes. Since the first reported primate ZIKV infection in 1947, sporadic human cases have occurred in Africa and Asia, followed by outbreaks in Micronesia and French Polynesia, culminating in widespread infection in the Americas in 2015–2016 [1–4]. In May 2015, locally transmitted infection in the Western Hemisphere was first reported in Brazil; the predominant strain was related to the Asian genotype [5]. ZIKV disseminated among this largely immunologically naïve population, where the World Health Organization estimates >534,000 confirmed or suspect cases, involving the majority of Western Hemisphere countries by the end of 2016 [6]. As of September 2017, 5,431 cases have been reported in the continental United States (U.S.), of which 5,155 were travel-associated, 225 were locally acquired mosquito-borne cases, and 36,644 cases were reported in Puerto Rico and the U.S. Virgin Islands [7].

ZIKV infection is often asymptomatic and usually self-limited, with most symptoms resolving in 7–10 days [4]. Patients typically present with rash, arthralgia, conjunctivitis, or fever [1, 3, 4]. More serious complications include congenital syndrome (microcephaly and other fetal abnormalities), Guillain-Barré syndrome (GBS) and other neurological disorders [8–23]. ZIKV is detectable for approximately 1 week in blood and 2 weeks in urine [24–26].

The Veterans Health Administration (VHA) has health care facilities throughout the U.S. and territories. We perform ongoing surveillance for emerging pathogens, and reported the first ZIKV case in Puerto Rico in December 2015 [27]. Since 46% of all U.S. Veterans and 62% of Veterans in Puerto Rico are aged ≥65 years and have significant comorbidities, they could be at higher risk for severe ZIKV infection compared to those in the general U.S. population exposed to the virus (i.e., returning travelers and those living in areas with local ZIKV transmission) [28–30]. Herein, we describe characteristics of ZIKV-infected Veterans and investigate risk factors for hospitalization and neurological complications.

## Methods

### Ethics statement

ZIKV testing and surveillance were conducted as part of VHA operations and public health activities. As such, the VHA Office of Research Oversight considers public health investigations as operational and not research in VHA [31]. Since only ZIKV-positive cases required reporting to public health and U.S. federal agency authorities, negative cases were not reviewed. Patient data were anonymized after data abstraction for analyses.

### Data collection and analysis

We identified patients from all VHA facilities with ZIKV test results for specimens collected between December 1, 2015–October 31, 2016, utilizing VHA national data sources. Additional case finding was performed by querying inpatient and outpatient encounter data for Zika-specific International Classification of Diseases, Clinical Modification, 10^th^ Revision (ICD-10-CM) code A92.5 and from VHA facility communications with VA leadership.

**Fig 1:**
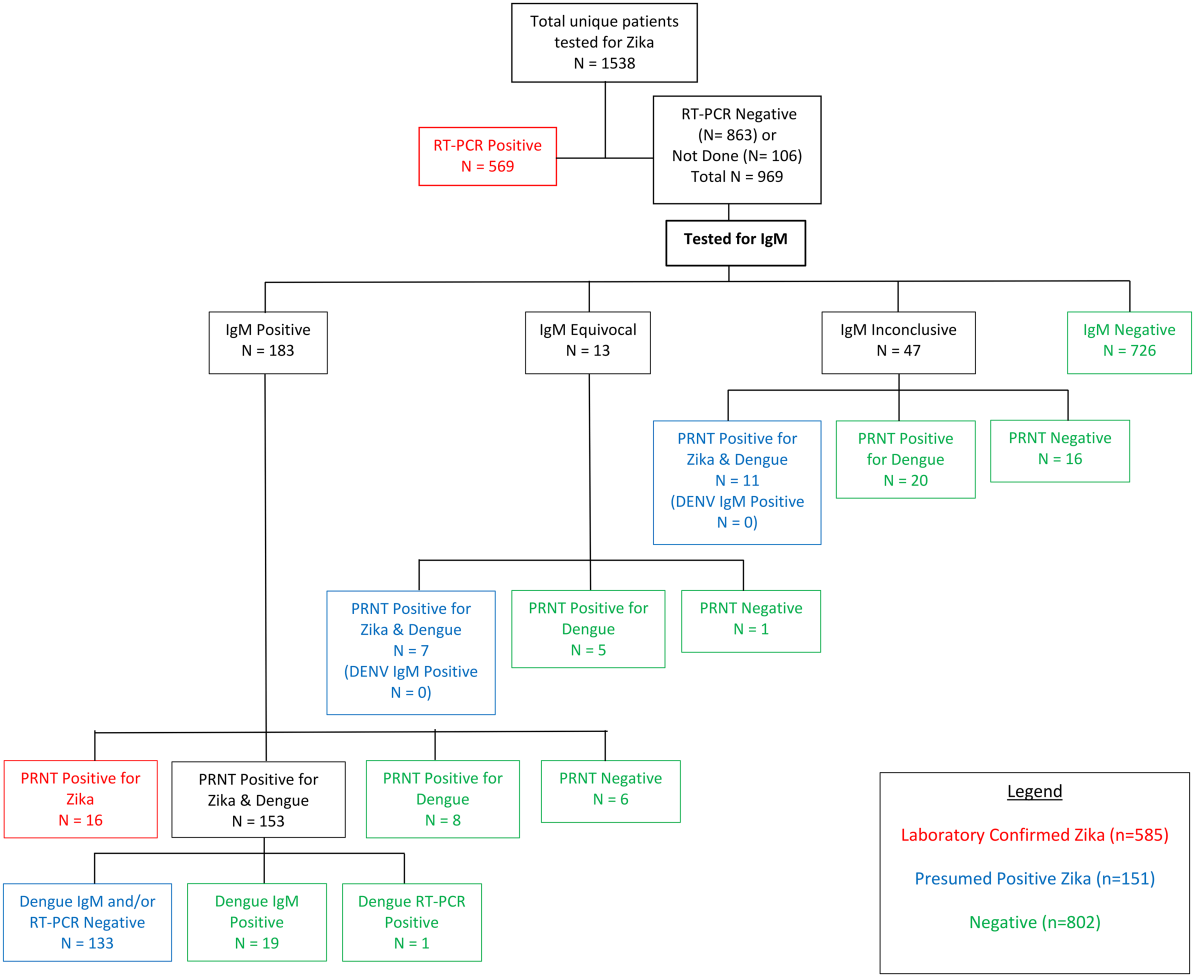
Zika virus flow diagram. RT-PCR, reverse-transcription polymerase chain reaction; IgM, immunoglobulin M; DENV, Dengue virus; PRNT, plaque reduction neutralization testing [25].

Testing for ZIKV was performed at the VHA’s Public Health Reference Laboratory (PHRL), and public health, federal or commercial laboratories. Testing and confirmation of ZIKV infection in our patient population is summarized in Fig 1. Initially, PHRL utilized a ZIKV reverse transcriptase PCR (RT-PCR) assay described previously [32]. Following U.S. Food and Drug Administration approval in March 2016, the Centers for Disease Control and Prevention (CDC) Trioplex RT-PCR assay for ZIKV, Dengue virus (DENV) and chikungunya virus (CHIKV) (in serum, whole blood, urine and spinal fluid) and ZIKV MAC IgM enzyme-linked immunosorbent assay (ELISA) (Anti-Zika Virus IgM Human MAC-ELISA kit, CDC) (for serum only) were used according to manufacturer’s recommendations [33, 34]. DENV (DENV *Detect*, InBios, Seattle, WA) and CHIKV (Abcam, Cambridge, MA) serum IgM ELISA assays were also performed per manufacturer’s recommendations. Testing methods performed by non-VA laboratories were unable to be confirmed. Samples with presumptive positive, equivocal or inconclusive ZIKV IgM results with an RT-PCR result that was either negative or not performed were sent to CDC for confirmatory testing using PRNT for DENV and ZIKV IgM [25]. Coinfection was defined as CHIKV and ZIKV-positive RT-PCR or IgM assays. In addition, patients with positive DENV RT-PCR and ZIKV RT-PCR assays would be considered coinfected. Potential cross-reaction of tests was defined as positive DENV IgM or RT-PCR and ZIKV IgM with plaque reduction neutralization test (PRNT) positive for DENV and ZIKV IgM results within 30 days of testing. Patients who were ZIKV RT-PCR and DENV IgM positive without PRNT were unable to be categorized as coinfected or cross-reactive. Since laboratory testing was performed based on clinician orders, not all assays were performed in all patients. In addition, CDC-recommended testing strategies changed over the course of 2016 [25, 26, 35].

For all patients with available ZIKV-positive diagnostic test results, we extracted demographics, clinical symptoms during acute illness and travel history from clinical notes, laboratory results (leucocyte, lymphocyte and platelet count; creatinine, alanine [ALT] and aspartate [AST] aminotransferases), concomitant medications (including 3-hydroxy-3-methylglutaryl-coenzyme (HMG-CoA) reductase inhibitors, antidiabetic, nonsteroidal anti-inflammatory drugs (NSAID), antidementia, oral glucocorticoids, antineoplastics, antivirals (for HIV-1 treatment), immunosuppressants, and intravenous immunoglobulin), hospitalizations, and outcomes from VHA electronic health records (EHR). Comorbidities were identified by extraction of discharge and encounter ICD-10-CM codes and provider notes. Age-adjusted Charlson co-morbidity indices (CCI) were calculated as a measure of a patient’s health status [36]). Laboratory cutoffs were as follows: leukopenia (<4,500 white blood cells [WBC]/μL); leukocytosis (>11,000 WBC/μL); lymphopenia (<1,000 lymphocytes/μL); and thrombocytopenia (<155,000 platelets/μL). Acute kidney injury (AKI) was calculated based on serum creatinine levels that were collected at presentation and the most recent serum creatinine prior to ZIKV infection (used as baseline). AKI was categorized into stage I (1.5–1.9 times baseline), stage II (2.0–2.9 times baseline), and stage III (≥3.0 times baseline) [37]. Abnormal hepatic function was determined by elevated transaminasemia 1–1.9 times upper limit of normal (ULN) (AST 34, ALT 40), 2–2.9 times ULN, and ≥3 times ULN [38]. Neurologic complications were determined by review of encounter ICD-10-CM codes after ZIKV diagnosis through 3/31/2017.

Positive cases were categorized as laboratory-confirmed which was defined as a patient with detectable ZIKV RNA by RT-PCR in serum or urine or a patient with positive ZIKV IgM ELISA result and confirmatory PRNT positive for ZIKV IgM only. A presumed positive case was a patient with a positive serum ZIKV IgM and negative DENV IgM result or not tested and a PRNT result positive for ZIKV and DENV IgM. Characteristics of patients with ZIKV infection diagnosed in VA Caribbean Health Care System (VACHCS) were compared to elsewhere in the U.S. In addition, factors associated with 1) hospitalization and 2) timing of diagnosis (in relation to infection) was assessed. Patients with a positive RT-PCR result for ZIKV (regardless of their IgM laboratory test results) were assumed to have been diagnosed earlier during their infection than patients with only an IgM-positive result for ZIKV (i.e., early vs. late where the latter was used as the referent group).

Student’s t-test and χ^2^ test were used to estimate associations between continuous and categorical variables, respectively. Logistic regression was used to estimate crude and adjusted odds ratios (OR) and 95% confidence intervals (CI) for factors associated with hospitalization and timing of diagnosis. For all clinical and medication-related data, “no” and “unknown” responses were combined as “no” and served as the referent group for all logistic regression models. A multi-stage backwards model building approach was used to develop a parsimonious main effects model (S1 Text and S1 Fig). That is, age group (in 10-year age categories), age-adjusted CCI, and the individual comorbidities used in the CCI were included in stage I. Non-significant comorbidities were removed from the model. Clinical findings, laboratory findings, and medications prescribed as an outpatient prior to ZIKV infection were added and subsequently removed (per non-significance) in stages II, III, and IV, respectively. Age group and age-adjusted CCI remained in the model, regardless of statistical significance until the completion of stage IV. The Kaplan-Meier log-rank test was used to estimate differences in length of stay. Non-parametric tests (e.g., Wilcoxon, Mann-Whitney) were used for non-normally distributed data (e.g., age among those who died). Death among patients with laboratory-confirmed ZIKV infection was analyzed in separate age-adjusted models. An alpha of 0.05 was used to determine statistical significance. Analyses were performed by using SAS 9.4 (SAS Institute, Inc., Cary, North Carolina).

## Results

We identified 1,538 VHA patients with ZIKV test results during December 2015– October 2016 (Fig 1). PHRL performed 1,424 (93%) of these tests and the remainder were performed at non-VA laboratories. Seven hundred thirty-six (48%) patients were RT-PCR-positive or serum IgM presumed positive confirmed with PRNT. Of these, 585 patients were laboratory-confirmed by RT-PCR (n = 569) or positive IgM with PRNT positive for ZIKV IgM only (n = 16). Per CDC guidelines and since there was a lack of active dengue cases seen by PCR or dengue specific IgM testing, the remaining 151 patients were presumed positive for ZIKV as their ZIKV IgM PRNT was positive for both ZIKV and DENV [24]. Demographic and clinical factors are summarized in Table 1; cumulatively, there were 655 (89%) male patients, with the majority (93%) of patients diagnosed at VACHCS, and the remaining 7% of patients diagnosed at 24 other VHA medical centers. Documented travel for those diagnosed in the continental U.S. included non-Puerto Rico Caribbean (18), Puerto Rico (16), Central America (9), South America (2), Indonesia (1), and Senegal (1). Six patients had exposure only in Florida. Mean age of all patients was 58.8 years (range 20–99). Patients from VACHCS versus returning travelers with ZIKV infection were older (mean age 60 versus 47 years; p< 0.001).

**Table 1:** Demographics and clinical characteristics of patients with ZIKV infection—Veterans Health Administration, December 2015-October 2016 (n = 736).

Characteristic	VACHCS n = 683 (92.8%)	United States^a^ n = 53 (7.2%)	p-value
**Age, mean ± SD, y**	59.7±16.6	46.7±11.9	<0.001
**Male, no. (%)**	617 (90)	38 (72)	<0.001
**Hispanic, no. (%)**	625 (92)	26 (49)	<0.001
**Race, no. (%)**			
White	599 (91)	27 (64)	<0.001
Black	55 (8)	13 (31)	
Asian/Pacific Islander	6 (0.9)	2 (5)	
Missing data	23	11	
**Comorbidities, no. (%)**			
Myocardial infarction	24 (4)	1 (2)	1.000
Congestive heart failure	50 (7)	0 (0)	0.042
Peripheral Vascular Disease	67 (10)	0 (0)	0.011
Cerebrovascular Disease	21 (3)	0 (0)	0.391
Dementia	44 (6)	0 (0)	0.066
Chronic Pulmonary Disease	10 (2)	0 (0)	1.000
Connective Tissue Disease	11 (2)	1 (2)	0.595
Ulcer Disease	9 (1)	0 (0)	1.000
Mild Liver Disease	51 (8)	4 (8)	1.000
Diabetes	223 (33)	10 (19)	0.038
Hemiplegia	13 (2)	1 (2)	1.000
Moderate to Severe Renal Disease	51 (8)	1 (2)	0.166
Diabetes with End Organ Damage	0 (0)	0 (0)	NA
Any Tumor/Leukemia/Lymphoma	36 (5)	2 (4)	1.000
Moderate to Severe Liver Disease	0 (0)	0 (0)	NA
Metastatic Solid Tumor	7 (1)	1 (2)	0.452
AIDS	6 (1)	0 (0)	1.000
**Charlson comorbidity index (age-adjusted) mean ± SD**	2.4±3.0	0.9±2.1	<0.001
**Zika virus Laboratory Testing (n %)**			
RT-PCR +	533 (78)	36 (68)	<0.001
IgM + and PRNT + for Zika only	8 (1)	8 (15)	
IgM + and PRNT + for Zika and Dengue	142 (21)	9 (17)	
**Highest Level of care received, no. (%)**			
Telemedicine	3 (0.4)	1 (2)	<0.001
Department of Health	0 (0)	1 (2)	
Rehabilitation	0 (0)	1 (2)	
Primary care provider	226 (33)	28 (53)	
Emergency department	363 (53)	19 (36)	
Hospitalized	71 (10)	3 (6)	
Intensive care unit	20 (3)	0 (0)	
**Died, no. (%)**	19 (3)	0 (0)	0.388
**Neurologic Complications**^b^	44 (6)	2 (4)	
Acute transverse myelitis in demyelinating disease of central nervous system	1	0	NA
Altered mental status	7	0	NA
Ataxia	3	0	NA
Atypical viral infection of CNS	0	1	NA
Convulsions	4	0	NA
Critical care illness polyneuropathy	2	0	NA
Cerebral vascular accident	11	0	NA
Demyelinating disease of CNS	1	0	NA
Encephalopathy	2	0	NA
Facial weakness following cerebral infarction	1	0	NA
Guillain-Barré syndrome	5	0	NA
Idiopathic progressive neuropathy	2	0	NA
Inflammatory polyneuropathy	2	0	NA
Meningitis	2	1	NA
Myelitis	2	0	NA
Myopathy	1	0	NA
Neuralgia and neuritis	3	0	NA
Non-traumatic intracranial hemorrhage	3	0	NA
Non-traumatic subarachnoid hemorrhage	1	0	NA
Other idiopathic peripheral autonomic neuropathy	1	0	NA
Polyneuropathy	7	0	NA
Seizures	3	0	NA
Transient ischemic attacks	3	0	NA
Tremors	2	0	NA
Viral encephalitis	3	0	NA
Visual hallucinations	1	0	NA

VACHCS, VA Caribbean Healthcare System; SD, standard deviation; PRNT, plaque reduction neutralization test; CNS, central nervous system; NA, not applicable

^a^Excluding Puerto Rico.

^b^Not mutually exclusive groups

Four hundred seventy-four of 736 (65%) patients presented to an emergency department. Most common documented symptoms in patients with ZIKV infection were arthralgia/myalgia 92%, rash 90%, conjunctivitis 75%, and reported fever 66% (Table 2). Documented fever and myalgia/arthralgia or rash was reported for 378 (51%) patients and subjective fever and rash for 315 (43%) patients.

**Table 2:** Documented symptoms, signs and laboratory abnormalities of patients with ZIKV infection.

Documented Symptoms	N^a^ (%)
Fever (reported)	419/640 (66)
Chills	162/343 (47)
Rash	552/612 (90)
Arthralgia/myalgia	490/535 (92)
Conjunctivitis	220/293 (75)
Headache	213/290 (73)
Neurological symptoms^b^	123/144 (85)
**Leukopenia**^c^	
On presentation	229/657 (35)
At nadir	241/657 (37)
**Leukocytosis**^d^	
On presentation	55/657 (8)
At peak	70/657 (11)
**Lymphopenia**^e^	
On presentation	178/648 (27)
At nadir	193/648 (30)
**Thrombocytopenia**^f^	
On presentation	131/657 (20)
At nadir	162/657 (25)
**Acute Kidney Injury**^g^	25/475 (5)
Stage I	20/475 (4)
Stage II	3/475 (0.6)
Stage III	2/475 (0.4)
**Hepatic Transaminitis**^h^	121/378 (32)
1–1.9 times ULN	80/378 (21)
2–2.9 times ULN	24/378 (6)
≥3 times ULN	17/378 (5)

ULN, upper limit of normal

^a^Documented symptoms and signs as recorded in medical records for patients during evaluation for ZIKV infection. Denominator includes all patients with documented symptom (present or not present) or laboratory test result (normal or abnormal).

^b^Neurologic symptoms included altered mental status, aphasia, back pain radiating to leg, blurred vision, burning sensation in leg, “decreased light tolerance”, difficulty walking, disorientation, dizziness, erratic behavior, falls, fall with head trauma, forgetfulness, gait problems, generalized weakness, hypoactive, numbness, slurred speech, syncope, tingling, weakness, worsening dementia

^c^Leukopenia, <4,500 white blood cells [WBC]/μL

^d^Leukocytosis, >11.000 WBC/μL

^e^Lymphopenia, <1,000 lymphocytes/μL

^f^Thrombocytopenia, <155,000 platelets/μL

^g^Acute kidney injury stage I, increase in serum creatinine by 1.5–1.9 mg/dL times baseline (last creatinine); stage II, 2.0–2.9 times baseline; and stage III, ≥3.0 times baseline

^h^ALT (alanine aminotransferase): 10 to 40 IU/L, AST (aspartate aminotransferase): 10 to 34 IU/L

The distribution of laboratory findings during their ZIKV illness is shown in Table 2. Among ZIKV-positive patients, and of those who had hematology and chemistry testing performed, at their nadir, 37% had leukopenia (median, 3,700 WBC/μL; range, 800–4,400), 30% had lymphopenia (median, 740 lymphocytes/μL; range, 0–990), and 25% had thrombocytopenia (median, 126,500 platelets/μL; range, 17,000–149,000). Eleven percent had leukocytosis (median, 15,000 WBC/μL; range, 11,000–39,200). Twenty-five (5%) patients had acute kidney disease and 121 (32%) patients had elevated serum transaminases.

Concomitant use of HMG-CoA reductase inhibitors was the most frequently observed medication class (232 [32%]), followed by antidiabetics (124 [17%]), NSAID (83 [11%]), antidementia (40 [5%]), glucocorticoids (11 [2%]), antineoplastics (8 [1%]), antivirals (5 [0.7%]), and immunosuppressants (3 [0.4%]). No patients received intravenous immunoglobulin.

At VACHCS, 91 (12%) of 683 patients were hospitalized with median acute care length of stay (LOS) of 6 days (range 1–214 days), including 20 (3%) who were admitted to intensive care (ICU) with median LOS of 4 days (range 1–30 days); at VHA hospitals elsewhere in the U.S., 3 of 53 (6%) returning travelers with known hospitalization status were hospitalized with median LOS of 4 days (range 1–6 days) but none were admitted to an ICU. The length of stay between these two groups was not significantly different (p = 0.13). The average age among hospitalized patients was higher among the 91 patients at VACHCS than the three returning travelers in the continental U.S. (75 vs. 60 years, p<0.001). Crude measures of association with hospitalization are shown in Table 3.

**Table 3:** Risk factors associated with hospitalization among patients with ZIKV infection.

	Hospitalization		
	Crude OR	95% CI	p-value
Age, per 10-year increase	2.3	1.9, 2.8	<0.001
**Comorbidities**			
CCI	1.6	1.5, 1.7	<0.001
Myocardial infarction	12.0	5.2, 27.6	<0.001
Congestive heart failure	14.6	7.8, 27.1	<0.001
Peripheral Vascular Disease	3.4	1.9, 6.1	<0.001
Cerebrovascular Disease	6.8	2.8, 16.6	<0.001
Dementia	21.0	10.6, 41.7	<0.001
Chronic Pulmonary Disease	3.0	0.76, 11.8	0.12
Connective Tissue Disease	7.2	2.3, 22.9	<0.001
Ulcer Disease	2.0	0.40, 9.6	0.40
Mild Liver Disease	1.4	0.65, 2.90	0.41
Diabetes	2.5	1.6, 3.8	<0.001
Hemiplegia	9.9	3.3, 29.1	<0.001
Moderate to Severe Renal Disease	9.1	5.0, 16.5	<0.001
Any Tumor/Leukemia/Lymphoma	7.4	3.7, 14.5	<0.001
Metastatic Solid Tumor	7.1	1.7, 28.8	0.006
AIDS	1.4	0.16, 11.9	0.77
**Documented Clinical Findings**^a^			
Fever (reported)	1.3	0.80, 2.0	0.32
Chills	2.7	1.7, 4.2	<0.001
Rash	0.10	0.06, 0.16	<0.001
Arthralgia/Myalgia	0.71	0.45, 1.1	0.12
Conjunctivitis	0.40	0.23, 0.72	0.002
Headache	0.99	0.61, 1.6	0.96
Neurological symptoms^b^	10.0	6.2, 16.0	<0.001
Guillain-Barré syndrome	33.9	7.2, 159.4	<0.001
**Laboratory Abnormalities**			
Leukopenia at presentation	0.19	0.10, 0.37	<0.001
Leukopenia nadir	0.31	0.18, 0.54	<0.001
Leukocytosis at presentation	16.1	8.7, 29.7	<0.001
Leukocytosis at peak	21.5	12.1, 38.3	<0.001
Lymphopenia at presentation	2.8	1.8, 4.4	<0.001
Lymphopenia nadir	4.6	2.9, 7.4	<0.001
Thrombocytopenia	6.4	4.0, 10.1	<0.001
Acute kidney injury^c^	21.4	7.8, 58.8	<0.001
Hepatic transaminitis^d^	2.7	1.6, 4.4	<0.001
**Medications Outpatient**			
HMG-CoA reductase inhibitors	1.3	0.85, 2.1	0.20
Antineoplastic	2.3	0.46, 11.6	0.31
Antivirals for HIV	1.7	0.19, 15.5	0.63
Antidementia	5.3	2.7, 10.4	<0.001
Glucocorticoids	4.0	1.1, 14.0	0.029
Immunosuppressants	3.4	0.31, 38.3	0.31
Antidiabetics	2.7	1.7, 4.4	<0.001
Anti-inflammatories	0	NA	NA
**Diagnosis by RT-PCR**	0.46	0.27, 0.73	0.001

OR, odds ratio; CI, confidence interval

^a^No and Unknown combined and serve as referent group.

^b^Neurologic symptoms included altered mental status, aphasia, back pain radiating to leg, blurred vision, burning sensation in leg, “decreased light tolerance”, difficulty walking, disorientation, dizziness, erratic behavior, falls, fall with head trauma, forgetfulness, gait problems, generalized weakness, hypoactive, numbness, slurred speech, syncope, tingling, weakness, worsening dementia

^c^Acute kidney injury stage I, increase in serum creatinine by 1.5–1.9 mg/dL times baseline (last creatinine); stage II, 2.0–2.9 times baseline; and stage III, ≥3.0 times baseline

^d^ALT (alanine aminotransferase): 10 to 40 IU/L, AST (aspartate aminotransferase): 10 to 34 IU/L

Adjusted ORs, 95% CI and p-values controlling for all significantly associated factors are presented in Table 4. The odds of hospitalization significantly increased with CCI, connective tissue disease, congestive heart failure, dementia, neurologic symptoms, GBS, leukocytosis, thrombocytopenia, AKI, and glucocorticoid steroid use within 30 days of ZIKV testing. Patients presenting with a rash were less likely to be hospitalized. In additional adjusted analyses reported in Table 4, only having rash, conjunctivitis, leukopenia or lymphopenia at presentation were significantly associated with a positive RT-PCR test.

**Table 4:** Adjusted associations between comorbidities, clinical findings, laboratory findings, and medication use and 1) hospitalization 2) neurologic complications 3) ZIKV RT-PCR diagnosis.

Risk Factor	OR_adj_	95% CI	p-value
**Hospitalization**			
CCI (age adjusted)	1.2	1.0, 1.3	0.010
Connective tissue disease	29.5	3.6, 244.7	0.002
Congestive heart failure	6.0	2.0, 18.5	0.002
Dementia	3.6	1.1, 11.2	0.028
Skin symptoms	0.29	0.13, 0.66	0.003
Neurological symptoms	3.9	1.7, 9.2	0.002
Guillain-Barré syndrome	21.9	1.9, 249.6	0.013
Leukocytosis	11.8	4.5, 31.0	<0.001
Thrombocytopenia	7.8	3.3, 18.6	<0.001
Acute kidney injury	28.9	5.8, 145.1	<0.001
Glucocorticoids outpatient	13.3	1.3, 133.0	0.027
**ZIKV RT-PCR vs. IgM Diagnosis**			
Skin symptoms	2.4	1.5, 3.7	<0.001
Conjunctivitis	1.6	1.0, 2.7	0.043
Leukopenia (at presentation)	1.8	1.1, 2.9	0.017
Lymphopenia (at presentation)	1.8	1.1, 3.0	0.02
**Neurologic Complications**			
Hospitalized	5.9	2.9, 12.2	<0.001
Cerebrovascular disease	4.9	1.7, 14.4	0.004
Dementia	2.8	1.2, 6.6	0.021

OR_adj_, adjusted odds ratio; CI, confidence interval

Forty-six (6%) patients with ZIKV infection (37 confirmed, 9 presumed positive) also had neurologic complications as summarized in Table 1. Five patients had cerebrospinal fluid (CSF) tested for ZIKV, all of whom had at least one of the identified neurologic complications. One patient with altered mental status, meningitis and viral encephalitis was positive for ZIKV by RT-PCR in CSF and serum. CSF findings for this patient were consistent with a viral etiology, demonstrating mild pleocytosis (WBC 12/cm^3^, 88% polymorphonuclear leukocytes, 12% lymphocytes) and normal CSF protein level (30.2 mg/dL). As shown in Table 4, neurologic complications were significantly more likely in patients with a prior history of cerebrovascular disease (CVD) and dementia as well as those who had been hospitalized.

Of 81 women with a positive ZIKV test, 50 were of childbearing age (18–52 years old) and four were pregnant at the time of infection. Two of these patients were from VACHCS and two were identified as returning travelers. Three patients delivered their babies (further details on the outcome of the babies is unknown) and one patient miscarried at 9.5 weeks.

Fourteen patients were positive for DENV IgM and ZIKV RT-PCR alone, of whom one was hospitalized. These 14 were unable to be categorized as coinfection or cross-reaction as they did not have PRNT performed. Three additional patients were positive for all three viruses (CHIKV IgM, DENV IgM and ZIKV RT-PCR), of whom one was hospitalized. Fifty-six patients (8%), all diagnosed at VACHCS, were positive for ZIKV (RT-PCR [n = 43] or IgM [n = 13]) and CHIKV IgM coinfection, of whom nine (16%) were hospitalized. In adjusted analysis, age was significantly associated with coinfection and arthralgia/myalgia was significantly less common in these patients. There was no increased risk of hospitalization or neurologic complications associated with coinfection.

Nineteen (3%) patients died post-ZIKV infection, all of whom presented to VACHCS with ZIKV related symptoms of which 16 were hospitalized (Table 5). Fourteen of 19 had viremia at presentation (Table 5). The mean age of ZIKV patients who died was 82 years (range, 50–99 years), compared to 59 years (range, 20–98 years) for those at VACHCS who survived (p<0.001). The mean time from ZIKV testing until death was 39 days (range 3–104 days). Eighteen (95%) had at least one CCI condition. Thus, it was difficult to determine whether ZIKV infection contributed to death or not.

**Table 5:** Post-ZIKV infection deaths—Veterans affairs caribbean healthcare system, 2016–2017.

Age	ZIKV Test Positive	Date of ZIKV Testing	Date of Death	Days between Test and Death	Details Regarding Death
92	RT-PCR	6/27/2016	6/30/2016	3	Respiratory failure with metabolic and respiratory acidosis, infectious diarrhea
99	RT-PCR	6/4/2016	7/11/2016	37	Advanced dementia, sepsis, aspiration pneumonia
86	RT-PCR	7/5/2016	8/3/2016	29	Sepsis, aspiration pneumonia, gastrointestinal bleed
94	IgM/PRNT positive for Zika/Dengue	8/3/2016	8/14/2016	11	End stage heart disease
61	RT-PCR	5/24/2016	8/28/2016	96	No details in the chart
71	RT-PCR	6/6/2016	9/18/2016	104	Streptococcus mitis sepsis, refractory myelofibrosis
94	RT-PCR	9/10/2016	9/23/2016	13	Acute decompensated congestive heart failure, healthcare acquired pneumonia, acute renal failure, subhepatic collection
50	RT-PCR	9/8/2016	9/29/2016	21	ZIKV infection resolved then reportedly had a fall (no further details in chart)
97	RT-PCR	10/5/2016	10/9/2016	4	Chronic obstructive pulmonary disease exacerbation, Pseudomonas bacteremia, hypernatremia, non-ST elevation myocardial infarction, gastrointestinal bleed
92	IgM/PRNT positive for Zika/Dengue	10/18/2016	10/19/2016	1	Arrived unresponsive, non-ST elevation myocardial infarction with cardiopulmonary arrest
60	RT-PCR	8/14/2016	11/10/2016	88	Abdominal perforation, decompensated congestive heart failure, healthcare acquired pneumonia, end stage renal disease
86	RT-PCR	8/27/2016	12/7/2016	102	Cardiac arrest, acute myocardial infarction, distributive shock, multi-organ failure
73	IgM/PRNT positive for Zika/Dengue	10/23/2016	12/11/2016	49	HIV-HCV co-infected patient with viral encephalitis, acute intracranial hemorrhage
87	IgM/PRNT positive for Zika/Dengue	5/2/2016	12/17/2016	229	Septic shock due to complicated urinary tract infection with *E*. *coli*, bacteremia with *E*. *coli*, *M*. *morganii*, and *E*. *faecalis* with obstructive right ureterolithiasis
85	IgM/PRNT positive for Zika/Dengue	7/28/2016	12/24/2016	149	Chronic obstructive pulmonary disease with respiratory failure, aspiration pneumonia, atrial fibrillation
82	RT-PCR	7/19/2016	1/20/2017	185	Acute decompensated congestive heart failure, acute on chronic kidney disease, diarrhea, pneumonia, hypertension
88	RT-PCR	6/23/2016	1/23/2017	214	Clostridium difficile sepsis, healthcare acquired pneumonia, respiratory failure, non-ST elevation myocardial infarction, discharged home but died suddenly at home after breathing treatment
74	RT-PCR	8/31/2016	1/27/2017	149	Non-resectable cholangiocarcinoma, biloma
81	RT-PCR	7/19/2016	3/2/2017	226	Advanced dementia, aspiration pneumonia

## Discussion

Our study is the first to characterize U.S. Veterans with ZIKV infection. Testing varied based on test availability, provider preference, or presenting symptoms. The majority received a diagnosis in Puerto Rico, although 53 were returning travelers or had locally acquired infection elsewhere in the U.S. Among returning travelers, three were hospitalized, whereas in Puerto Rico, where patients were older and had more comorbidities, approximately 13% of patients with ZIKV infection were hospitalized, of whom 3% were admitted to ICU, and 3% died post ZIKV infection. Although we cannot directly link the deaths with ZIKV, the number of deaths was higher among VHA patients compared with a report from Puerto Rico in December 2016 that described only 5 deaths identified by surveillance on the island [39]. CCI was associated with increased risk for hospitalization which was possibly related to a lower threshold for hospitalization in those with significant chronic illness. After adjusting for CCI, connective tissue disease, CHF, dementia as well as presenting with neurologic symptoms, leukocytosis, thrombocytopenia, AKI or being prescribed glucocorticoids 30 days prior to ZIKV diagnosis was associated with increased risk for hospitalization. However, presenting with a rash made hospitalization less likely and no patients receiving a NSAID were hospitalized.

Hospitalization and deaths are reported to be uncommon in ZIKV infection [3, 40–44]. During the 2007 ZIKV outbreak in Micronesia, among 49 confirmed and 59 probable cases, patients presented with typical symptoms described here, but none were hospitalized and none died [3]. Although patients in that study were on average >10 years younger and fewer had comorbidities than U.S. Veterans. In Brazil, among 119 ZIKV confirmed patients only one hospitalization and no deaths were reported [41]. Hospitalizations and death (<1%) were noted in Puerto Rico from November 2015–July 2016 [40]. In our Veteran population, 3% died after a ZIKV diagnosis and 13% were hospitalized which is higher than other ZIKV studies and may be related to Veterans increased comorbidities [30].

Several studies have documented coinfection with ZIKV and CHIKV [45–47]. A prior study identified patients from Nicaragua with positive CHIKV and ZIKV [47]. Since cross-reaction is unlikely between these viruses, these patients were noted to have coinfection. In their study, 16/263 (6%) ZIKV-positive patients were noted to have coinfection with CHIKV and ZIKV [47]. In the Nicaraguan cohort, patients with coinfection trended toward more hospitalization and had similar symptoms to those monoinfected [47]. In our study, 56/736 (8%) patients were identified as being positive for ZIKV and CHIKV IgM (with or without positive DENV IgM). No patients were identified with ZIKV and CHIKV or DENV by RT-PCR. In patients with coinfection, there was no increased risk in hospitalization or neurologic complications but there was an increased risk of coinfection with advanced age. Symptoms were similar between groups except there was a decrease in documented arthralgia/myalgia in coinfected patients.

ZIKV has been documented to have congenital as well as neurologic complications [8–23]. Forty-six patients in our cohort were also noted to have neurologic complications after ZIKV infection. While these neurologic complications are quite broad, they identify potential complications post-ZIKV infection. Prior history of CVD and dementia as well as being hospitalized with ZIKV increased risk of neurologic complication. It was difficult to confirm whether these other neurologic complications were the result of ZIKV infection. Since neonatal and pediatric care was not provided by VA, the status of the infants exposed to ZIKV is unknown.

There are several limitations to our retrospective study. Cases not tested or with results not documented in VA’s EHR could not be identified; asymptomatic and mild cases were unlikely to have testing performed; early dated cases were not tested for ZIKV IgM as it was not available at the time of clinical testing, so some cases that were RT-PCR negative may have been missed; samples from VACHCS prior to December 2015 that were tested for DENV and CHIKV were not tested for ZIKV; samples from facilities in the continental U.S. were only tested for those ordered by the provider and complete testing may not have been ordered by provider depending on timing of symptoms and possible exposure. Some health departments had strict testing criteria and submitted Veteran samples may have been rejected or not tested. Across the VA system, and specifically at VACHCS, testing was not restricted, particularly for testing performed in VA, as there was an ongoing outbreak. We were unable to determine if deaths or neurologic complications were directly related to ZIKV infection. No ICD-10-CM diagnosis code was available for ZIKV until 10/1/2016 for additional case identification purposes. Medications not obtained within the VA could not be identified. Patients receiving care outside of the VA were unable to be reviewed for neurologic complications. Only Veterans who presented to VHA facilities and had appropriate diagnostic testing completed were included. Since asymptomatic individuals were unlikely to be tested for ZIKV, overall burden of disease was unable to be determined. Given the nature of the investigation, primary focus was placed on ZIKV-positive patients. Sample sizes among certain subgroups limited inferences from statistical analyses. Veterans represent a unique group of patients who tend to have increased age and comorbidities compared to the general population [30]. Among returning travelers, many of whom presented for care in the U.S. during the convalescent period, when diagnosis is dependent upon serology, some diagnoses could have been missed as ZIKV IgM typically declines after several weeks to months [26].

Clinicians practicing in areas with ZIKV transmission should be aware that ZIKV infection among elderly patients and patients with comorbidities, including connective tissue disease, dementia and CHF, those on glucocorticoids, and those presenting with neurologic symptoms, leukocytosis, AKI, and thrombocytopenia may have more severe disease. In addition, patients hospitalized and those with prior history of CVD and dementia were more likely to have neurologic complications.

Larger studies are required to determine risks associated with atypical complications, intensive care utilization and death associated with ZIKV infection; and whether prevention strategies or closer monitoring for those at greatest risk for such complications after ZIKV infection should be targeted.

## Supporting information

S1 Text Statistical methods. (DOCX)

S1 Fig Flow diagram of logistic regression analysis to assess associations with hospitalization status among cases of Zika virus. (DOCX)
