# Supplementary material for: Zika virus infection in the Veterans Health Administration (VHA), 2015-2016
Source: PLoS Negl Trop Dis. 2018 May 24;12(5):e0006416. doi: 10.1371/journal.pntd.0006416 (PMC5967711; doi:10.1371/journal.pntd.0006416)
Supplement: S1 Text — (DOCX) [file pntd.0006416.s001.docx]

**S1 Text. Statistical Methods.**

Independent variables were grouped in to demographic, comorbid conditions, clinical findings, laboratory findings, medications prescribed in an outpatient setting, and other potentially associated factors. The demographic variable of interest was age group (in 10-year categories). The comorbid conditions are those used in the calculation of the Charlson Comorbidity Index (CCI): myocardial infarction, congestive heart failure, peripheral vascular disease, cerebrovascular disease, dementia, chronic pulmonary disease, connective tissue disease, ulcer disease, mild liver disease, moderate to severe liver disease, hemiplegia, diabetes, diabetes with end organ damage, moderate to severe renal disease, any tumor/leukemia/lymphoma, metastatic solid tumor, and AIDS. In addition, the age-adjusted CCI was included with the variables in the comorbid conditions list. The clinical findings included reported fever, chills, rash, arthralgia/myalgia, conjunctivitis, headache, neurological symptoms, and Guillain-Barré syndrome (GBS). Laboratory findings included leukopenia (on presentation and at nadir), leukocytosis (on presentation and at peak), lymphopenia (on presentation and at nadir), thrombocytopenia (on presentation and at nadir), acute kidney injury, and hepatic transaminitis. Medications prescribed in an outpatient setting prior to the Zika virus infection diagnosis included HMG-CoA reductase inhibitors, antineoplastics, antivirals for HIV infection, antidementia, glucocorticoids, immunosuppressants, antidiabetics, and anti-inflammatories. The other potentially associated factors were also used as dependent variables in unique analyses: hospitalization status, diagnostic method type (i.e., ZIKV RT-PCR vs. IgM diagnosis), and having a post-neurologic complication.

A multi-stage backwards model building approach was used to develop a parsimonious main effects model. That is, age group, age-adjusted CCI, and the individual comorbidities (n=18) used in the CCI were included in stage I. Non-significant comorbidities (n=14) were removed from the model, leaving age group and four comorbidity variables (i.e., age-adjusted CCI, connective tissue disease, congestive heart failure, and dementia) in the model. In stage II, the eight clinical findings variables were entered into the model, of which three were statistically significant (i.e., skin symptoms, neurological symptoms, and GBS) and thus remained in the model. In stage III, the nine laboratory findings variables were entered into the model, of which three were statistically significant (i.e., leukocytosis, thrombocytopenia, and acute kidney injury). In stage IV, the eight medications prescribed as an outpatient prior to ZIKV infection were added, of which only one was statistically significant (i.e., glucocorticoids). Age group and age-adjusted CCI remained in the model, regardless of statistical significance until the completion of stage IV. The analysis in which hospitalization status as the outcome of interest is and the flow sequence is illustrated in Figure S1. The same process was used when diagnostic method type (i.e., ZIKV RT-PCR vs. IgM diagnosis) and post-neurologic complications were used as the outcome of interest (as reported in Table 4).

In addition to assessing the comorbidity, clinical, laboratory, and medications variables as independent variables in the logistic regression models, we also assessed diagnostic method type and post-neurologic complications for potential association with hospitalization status. Hospitalization status and post-neurologic complications were assessed for potential association with diagnostic method type, and hospitalization status and diagnostic method type were assessed for potential association with post-neurologic complications in the final step of the model building process.
