# Supplementary material for: Zika virus infection in the Veterans Health Administration (VHA), 2015-2016
Source: PLoS Negl Trop Dis. 2018 May 24;12(5):e0006416. doi: 10.1371/journal.pntd.0006416 (PMC5967711; doi:10.1371/journal.pntd.0006416)
Supplement: S1 Fig — (DOCX) [file pntd.0006416.s002.docx]

**S1 Fig. Flow Diagram of Logistic Regression Analysis to Assess Associations with Hospitalization Status Among Cases of Zika Virus.**

Age group, 4 comorbidity, 3 clinical, and 3 laboratory variables enter next stage

Age group, 4 comorbidity, and 3 clinical variables enter next stage

Age group and 4 comorbidity variables enter into next stage

Diagnostic method type and post-neurologic complications individually assessed for association

*Comorbid factors: myocardial infarction, congestive heart failure, peripheral vascular disease, cerebrovascular disease, dementia, chronic pulmonary disease, connective tissue disease, ulcer disease, mild liver disease, moderate to severe liver disease, hemiplegia, diabetes, diabetes with end organ damage, moderate to severe renal disease, any tumor/leukemia/lymphoma, metastatic solid tumor, AIDS, and CCI.

**Clinical findings: reported fever, chills, rash, arthralgia/myalgia, conjunctivitis, headache, neurological symptoms, and Guillain-Barré syndrome.

†Laboratory findings: leukopenia (on presentation and at nadir), leukocytosis (on presentation and at peak), lymphopenia (on presentation and at nadir), thrombocytopenia (on presentation and at nadir), acute kidney injury, and hepatic transaminitis.

‡Outpatient medications: HMG-CoA reductase inhibitors, antineoplastics, antivirals for HIV infection, antidementia, glucocorticoids, immunosuppressants, antidiabetics, and anti-inflammatories.
